# Characterization, mapping and distribution of the two XMRV parental proviruses

**DOI:** 10.1186/1742-4690-8-S2-P10

**Published:** 2011-10-03

**Authors:** Oya Cingöz, Tobias Paprotka, Krista A Delviks-Frankenberry, Wei-Shau Hu, Vinay K Pathak, John M Coffin

**Affiliations:** 1 Department of Molecular Biology and Microbiology, Genetics Program, Tufts University, Boston, MA 02111, USA; 2 Viral Mutation Section, HIV Drug Resistance Program, National Cancer Institute, Frederick, MD 21702, USA; 3 Viral Recombination Section, HIV Drug Resistance Program, National Cancer Institute, Frederick, MD21702, USA

## Background

Xenotropic murine leukemia virus-related virus (XMRV) was previously reported to be associated with human prostate cancer and chronic fatigue syndrome. Recent work from our groups showed that XMRV was created through recombination between two endogenous murine retroviruses, PreXMRV-1 and PreXMRV-2, and that the recombination event that gave rise to XMRV occurred during the passaging of a prostate tumor xenograft in nude mice to generate the 22R*v1* cell line.

## Materials and methods

Multiple approaches that led to the identification of PreXMRV-2, as well as the distribution of both parental proviruses among different mouse species are described. The chromosomal loci of both PreXMRV-1 and PreXMRV-2 were mapped to the mouse genome, and the integration site information was used to analyze the distribution of both proviruses in 48 laboratory mouse strains and 46 wild-derived strains. Mouse strains found to carry both proviruses were analyzed for their Xpr1 receptor allele status by PCR.

## Results and conclusions

The strain distributions of PreXMRV-1 and PreXMRV-2 are quite different, the former being found predominantly in Asian mice and the latter in European mice, making it unlikely that the two XMRV ancestors could have recombined independently in the wild to generate an infectious virus. XMRV was not present in any of the 94 mouse strains tested, and among the wild-derived mouse strains analyzed, not a single mouse carried both parental proviruses. Interestingly, PreXMRV-1 and PreXMRV-2 were found together in three laboratory strains, Hsd nude, NU/ NU and C57BR/cd, consistent with previous data that the recombination event that led to the generation of XMRV could only have occurred in the laboratory. The three laboratory strains, as well as the mouse cells associated with the xenografts, carried the Xpr1^n^ receptor variant non-permissive to XMRV and X-MLV infection, suggesting that the presence of the xenografted human prostate tumor cells was required for the resulting XMRV recombinant to infect and propagate.

